# Clinical and behavior characteristics of individuals who used ketamine

**DOI:** 10.1038/s41598-022-04832-9

**Published:** 2022-01-17

**Authors:** Tony Szu-Hsien Lee, Yi-Hsuan Liu, Yun-Ju Huang, Wai-Kwong Tang, Yifan Wang, Sien Hu, Ching-Po Lin, Chiang-Shan Ray Li, Chia-Chun Hung

**Affiliations:** 1 Department of Health Promotion and Health Education, National Taiwan Normal University, Taipei, Taiwan; 2 Bali Psychiatric Center, Ministry of Health and Welfare, New Taipei City, Taiwan; 3 CTBC Center for Addiction Prevention and Policy Research, National Taiwan Normal University, Taipei, Taiwan; 4 Institute of Neuroscience, National Yang Ming Chiao Tung University, Taipei, Taiwan; 5 Department of Psychiatry, The Chinese University of Hong Kong, Hong Kong, China; 6 Department of Psychiatry, School of Medicine, Yale University, New Haven, CT USA

**Keywords:** Medical research, Public health, Addiction

## Abstract

This study aims to depict and compare clinical characteristics and risk behavior among groups of individuals using ketamine, polydrugs or smoking cigarette. A total of 185 drug-using participants and 49 smokers participated in this study. A cross-sectional interview was used to collect information on demographics, drug- and sex-related behaviors, HIV serostatus, lower urinary tract symptoms (LUTS), behavioral dispositions. N-back memory test was used to measure short-term memory. Result shows that 10 participants (5.41%) were HIV positive and 14 (7.57%) having LUTS. Individuals with ketamine and polydrugs use have significantly worse drug-related problem than cigarette smokers. Compared to cigarette smokers and ketamine users, individuals with polydrug users scored significantly higher on impulsivity measures. Cigarette smokers performed significantly better than the other two groups on the memory tests. A few patients had been infected with HIV and diagnosed with LUTS. Findings support that memory on short term recalls of patients with ketamine use might be impaired. Study findings warrants the necessarily of further study on influences of using ketamine.

## Introduction

Ketamine, a non-competitive NMDA receptor antagonist, was originally developed as analgesia and sedative for clinical use in the 1960s, but it evolved into a commonly abused drug among youths a few decades later. Following a surgical procedure, patients treated with ketamine reported that it induces schizophrenic-like symptoms such as delusions, dissociative sensations, emotion blunting and hallucination^1^. These mental side effects have limited the use of ketamine in medical settings but led to its recreational use^2^. The nonmedical use of ketamine has sharply increased in Asian countries^3,4^, along the emergence of associated physical and mental problems^5–7^.

Recreational use of ketamine in the United States began in the 1960s, soon after the drug was introduced. By 1980 it had spread worldwide, becoming a “club drug” with the expansion of rave culture^2,8^. Since ketamine is not included in the United Nations conventions of scheduled drugs, its prevalence has been under-reported. However, ketamine abuse has been found in studies of rave parties, night clubs, and peer gatherings in New York, the UK, continental Europe, Hong Kong, Mainland China, Malaysia and Taiwan^4,9^. During the last two decades, ketamine has grown to become one of the leading drugs sniffed by the youth population in many parts of Asia for its dissociative properties^10^. In Hong Kong, Ketamine was found to be the single most abused drug in 2006, whereas 20 years before its use there was less than 1%^11,12^. In previous studies, most ketamine users reported injecting other drugs in conjunction with it^2,12,13^. A study of 40 injected-drug users under 25 years old in New York City found that among frequent ketamine injectors, ketamine was the first drug they sampled; they enjoyed its effects, they were stably housed, and they associated with others who also injected ketamine^14^. A more recent study of 39,178 recreational ketamine users in Taiwan from 2009 to 2016 found that the 3-year standardized mortality ratio of unnatural deaths was 7.6 (95% CI = 6.7–8.6)^15^.

Today, the non-medical use of ketamine has been identified as an important public safety and health issue in some societies since it impacts a wide range of functions, especially when administered with other illicit drugs^16^. Although there may be benefits of using a very low dose of ketamine to treat patients with refractory depression^8,17^, misuse or chronic use of ketamine without a physician’s prescription can damage an individual’s health. Previous studies have found the consumption of ketamine to be associated with decreased frontal gray matter volume^18,19^, craving and neurocognitive impairment^1,20,21^, ulcerative cystitis^7,22^, and accidental deaths^23^. A study conducted in Mainland China showed that significantly less connectivity of the thalamic nuclear groups with the prefrontal cortex, the motor cortex/supplementary motor area, and the posterior parietal cortex in ketamine users compared to non-ketamine users^24^. Because ketamine is a club drug that can harm executive functions, its users are likely to engage in high-risk behavior that can lead to HIV infection^25,26^ and reckless driving^11^. Two studies found that frequent ketamine use and any ketamine use were associated with sexually risky behavior^25,27^. Young adults under the influence of various club drugs were shown to be at greater risk than non-users of engaging in dangerous behavior such as unprotected sex, thus facilitating the transmission of infectious diseases^26^. Collectively, these studies highlight the many adverse outcomes of ketamine misuse.

The drawback of these studies is that most of the ketamine users were also polydrug users, and thus the clinical consequences of any one of these drugs is difficult to assess^1^. To address this problem, in the present study we compared the clinical and behavioral characteristics of individuals who used ketamine only to polydrug users and to cigarette smokers who did not use any illicit drugs.

## Methods

### Ethical approval

The study protocol was reviewed and approved by the Human Subject Protection Committee of the Tri-Service General Hospital in Taiwan (A-1-102-05-004). All procedues were performed in accordance with the relevant guidelines and regulations.

### Participants

Study purpose and procedure were explained to all patients seeking non-opiate drug treatment in a hospital in New Taipei City, Taiwan between November 2015 and October 2016. Informed consent forms were obtained from 285 patients who agreed to participate. As for inclusion criteria, ketamine and polydrug user had to greater than 18 years of age but less than 35. On a urine test developed by Firstep Bioresearch, Inc. that assesses the presence of ketamine as well as methamphetamines, ecstasy, marijuana, and oxycodone, ketamine users had to test positive for ketamine and negative for the other drugs, whereas polydrug users had to test positive for ketamine and positive for at least one of the other drugs. Urine testing was done immediately after the collection of the consent form. Exclusion criteria for these two groups were (a) serious brain damage, (b) medical illness, and (c) pregnancy. Cigarette smokers age 18 or over were recruited through posters in hospitals to serve as controls, and they had to test negative on the urine test for all the drugs. Cigarette smokers were chosen as the control group because ketamine is ingested mostly by blunt smoking with cigarette in Taiwan. Each participant was reimbursed with 500 New Taiwan Dollars.

### Demographic and background information

Demographic and background information consisted of age, sex, education, employment, HIV serostatus, sexual preference, whether the person had been diagnosed as having a lower urinary tract disease (LUTD), as well as a history of drug and alcohol use. Objective assessments consisted of ASI and N-back memory. Subjective measures included nicotine dependence, impulsivity, sensitive to reward and punishment, aggression and psychiatric symptoms.

### Addiction severity index

The ASI was administered by a psychiatrist as a semi-structured interviewed to evaluate use of ketamine, alcohol, as well as other associated conditions^28^. Detailed information of ketamine use was also collected on the duration and frequency of use each substance and its quantity. HIV risk behavior was assessed by asking participants whether they had sex in the past 30 days and, if so, whether they used condoms, the number of sexual partners, and whether they exchanged sex for money or drugs. Finally, separate composite scores for the alcohol and drug items were calculated.

### Fagerström test for nicotine dependence

The FTND, also called the Fagerström Test for Cigarette Dependence, is a widely used measure of nicotine dependence^29^. It is a six-item questionnaire with scores ranging from 0 (no dependence) to 10 (highest dependence level) and focuses on core dependence criteria, including heavy use/tolerance and withdrawal^30^.

### Barratt impulsiveness scale-11^31^

The Chinese version of the BIS-11 was used to cover aspects of personality structures that are associated with drug and alcohol misuse. It is focused on impulsivity, harm avoidance, and sensation seeking. The Chinese version was shown to have acceptable reliability and validity in a sample of 720 high school students in Taiwan^32^.

### Buss–Perry aggression questionnaire

The BPAQ is a 29-item questionnaire in which participants rate statements along a 5-point continuum from “extremely uncharacteristic of me” to “extremely characteristic of me”. The scores are normalized on a scale of 0 to 1, with 1 representing the highest level of aggression^33^.

### Sensitivity to punishment/sensitivity to reward questionnaire

The SPSRQ is a self-report instrument that includes 48 yes/no questions divided into two subscales: Sensitivity to Reward (SR) and Sensitivity to Punishment (SP). SR, developed by Carver and White^34^, assesses the behavioral activation system (reward sensitivity), and SP, developed by Torrubia et al.^35^, assesses the “flight, fight and freezing” system (punishment sensitivity). Both subscales are based on predictions from a theory developed by Jeffrey Gray in 1976^36^.

### SCL-90

We used this self-report instrument to assess patients’ mental health. Reliability was assessed by alpha coefficients, which were found to range from 0.68 to 0.88 across subscales and the intra-correlations of each subscale ranged from 0.55 to 0.81^37^.

### N-back memory test

Developed more than 50 years ago^38^, the NBMT is an example of a popular methodological paradigm used to assess working memory in functional neuroimaging experiments. The test has respondents monitor the identity or location of a series of verbal or nonverbal stimuli and to indicate when the currently presented stimulus is the same as one presented earlier in the sequence. Performance under various working memory loads is determined by measuring reaction times and accuracy under different sequence lengths (Ns) between the two targets presentations. The most common Ns are 1-back, 2-back, and 3-back, with a 0-back frequently presented as the baseline. In this study, we used 0-, 1- and 2-back regimens, each presented three times in a random sequence of stimuli. The test was presented by computer.

### Statistical analysis

SPSS 23.0 was used to transform the data and perform the statistical analyses. Analysis of variance (ANOVA) and chi-square tests were performed to examine differences on each measure between the Ketamine, Polydrug, and Cigarette groups. The alpha criterion for significance was set at 0.05, two-sided, and the Bonferroni correction was applied to adjust for multiple comparisons. The Tukey test was used to test for significant post-hoc differences. A repeated measures ANOVA was performed to test whether there was a significant difference in short-term memory across groups.

## Results

The sample consisted of 159 patients with ketamine use, 26 patients with polydrug use (ketamine with methamphetamine and/or ecstasy) and 49 cigarette smokers. Table 1 presents the demographic and background statistics for the Ketamine, Polydrug, and Cigarette groups. For the total sample, the mean age was 24.35 years, 85.9% were male, mean education was 11.74 years, 80.7% were employed, and 92.3% were heterosexual. Of the 185 participants in the Ketamine and Polydrug groups, 10 (5.41%) were HIV positive and 14 (7.57%) had been diagnosed as having LUTS. For the Cigarette group, the mean nicotine dependence score on the FTND was 5.88 out of a possible 10. No significant differences between groups were found on the background variables.

**Table 1:** Background information of patients with Ketamine use, polydrug use and cigarette smoking.

Variables	Ketamine (n = 159)	Polydrug (n = 26)	Cigarette (n = 49)	Total (n = 234)	F/χ^2^	*p*
	Mean (SD) or n (%)	Mean (SD) or n (%)	Mean (SD) or n (%)	Mean (SD) or n (%)		
**Demographics**						
Age	24.39 (3.43)	24.50 (3.29)	24.16 (3.06)	24.35 (3.33)	0.11	.89
Sex					0.98	.36
Male	138 (86.8)	23 (88.5)	40 (81.6)	201 (85.9)		
Female	21 (13.2)	3 (11.5)	9 (18.4)	33 (14.1)		
Education	11.77 (1.91)	11.23 (1.39)	11.90 (0.51)	11.74 (1.66)	1.51	.23
Employed	126 (79.2)	219 (76.0)	43 (87.8)	188 (80.7)	2.14	.34
**Sexual preference**					5.13	.08
Heterosexual	151 (95.0)	23 (88.5)	42 (85.7)	216 (92.3)		
Homo/Bi-sexual	8 (5.0)	3 (11.5)	7 (14.3)	18 (7.7)		
HIV positive	9 (5.7)	1 (3.8)	0 (0)	10 (5.41)	4.97^a^	.08
LUTS	11 (6.9)	3 (11.5)	0 (0)	14 (7.57)	7.42^a^	.02
Cigarette dependence (FTQ)	6.08 (2.21)	5.65 (2.00)	5.37 (2.63)	5.88 (2.29)	1.99	.14

There was none in the cigarette group with HIV or LUTS.

*LUTS* Lower urinary tract symptoms.

^a^Fisher’s exact test.

Results for drug-related and sex-related risk behavior are presented in Table 2. With respect to drug-related risk behavior, 175 (94.6%) used Ketamine by smoking joints and 10 (5.4%) by snorting. The mean numbers of days using Ketamine in the past 30 days were 14.41 and 18.50 for the Ketamine and Polydrug groups respectively. The mean duration of their use of Ketamine was 43.98 months, and the mean dose was 0.46 g. The mean ASI composite scores for alcohol and drug problems were 0.150 and 0.059 for the Ketamine and Polydrug groups respectively. There is a significant difference in the average ASI drug composite scores across the three groups, F(2, 214) = 39.15, *p* < 0.001. Post hoc Tukey tests showed that the Polydrug group had significantly more severe drug problems than the Ketamine group (*p* = 0.001), and the Ketamine group had significantly more severe drug problems than the Cigarette group (*p* < 0.001).

**Table 2:** Drug use and sex risk behavior amongst participants.

Variables	Ketamine (n = 159)	Poly drugs (n = 26)	Cigarette (n = 49)	Total (n = 234)	F/χ^2^	*p*
	Mean (SD) or n (%)	Mean (SD) or n (%)	Mean (SD) or n (%)	Mean (SD) or n (%)		
**Drug-related variables**						
Ketamine use routes					2.23	.136
Smoking	152 (95.6)	23 (88.5)		175 (94.6)		
Snorting	7 (4.4)	3 (11.5)		10 (5.4)		
Days used in past 30 days	13.74 (11.18)	18.50 (10.60)		14.41 (11.19)	4.11	.044
Ketamine use duration (months)	43.46 (42.88)	47.04 (44.85)		43.98 (43.06)	0.15	.697
Ketamine dose used per time (g)	0.44 (0.54)	0.58 (1.28)		0.46 (0.69)	0.99	.321
Addiction severity index						
Alcohol composite score	0.160 (0.151)	0.073 (0.091)	0.158 (0.118)	0.150 (0.141)	4.45	.013
Drug composite score	0.069 (0.059)	0.112 (0.074)	0.003 (0.014)	0.059 (0.063)	39.15*	.000
**Sex-related risk**						
Multiple sexual partners					2.19	.335
No	121 (76.1)	18 (69.2)	41 (83.7)	180 (76.9)		
Yes	38 (23.9)	8 (30.8)	8 (16.3)	54 (23.1)		
Exchanging drugs/money/sex 30 days					4.95	.084
No	146 (91.8)	23 (88.5)	49 (100.0)	218 (93.2)		
Yes	13 (8.2)	3 (11.5)	0 (0)	16 (6.8)		
Condom use in 6 months					4.00	.676
Did not use	20 (12.6)	3 (11.5)	4 (8.2)	27 (11.5)		
Occasionally	32 (20.1)	7 (26.9)	13 (26.5)	52 (22.2)		
Often	21 (13.2)	4 (15.4)	3 (6.1)	28 (12.0)		
Always/no sex	86 (54.1)	12 (46.2)	29 (59.2)	127 (54.3)		

Bonferroni correction was used to adjust multiple comparisons. Tukey’s method was performed to test the post-hoc difference.

*p < .05/9 = .0056.

As for sex-related risk behavior on the ASI, 54 (23.1%) of participants had multiple sexual partners in the last 30 days, and 16 (6.8%) exchanged drugs for sex or money. During the previous 3 months, 27 (11.5%) did not use condoms at all, 52 (22.2%) used them occasionally, 28 (12.0%) used them often, and 127 (54.3%) always used them or did not have sex. The ANOVA did not reveal significant differences across the three groups for sex-related risk behavior.

Table 3 presents results for behavioral dispositions on the BIS-11 and for short-term memory on the NBMT. The mean BIS-11 scores for impulsivity, aggression, and sensitivity to reward and punishment were 68.57, 69.60, 12.03 and 10.48, respectively. A significant difference was found in impulsivity scores across the three groups, F(2, 229) = 9.27, *p* < 0.001. Post-hoc results from the Tukey tests showed that the mean BIS-11 score was significantly higher for the Polydrug group than for the Ketamine (*p* = 0.02) and Cigarette group (*p* = 0.00), and significantly higher for the Ketamine group than for the Cigarette group (*p* = 0.01). No significant difference was found in scores of BPAQ, SP and SR. As for short-term memory, results from the repeated ANOVA showed that participants in the Cigarette group performed significantly better on the NBMT than the Ketamine group, *F*(2, 231) = 5.78, *p* = 0.004. Post hoc results from the Tukey tests indicated that accuracy of the NBMT was higher for the Cigarette group than the Ketamine (*p* = 0.002), and the Polydrug group (*p* = 0.002). As shown in Table 4, the differences across the three groups in mental health as measured by the SCL-90 were not significant.

**Table 3:** Behavioral dispositions and short term memory performance of participants.

Variables	Ketamine (n = 159)	Poly Drugs (n = 26)	Cigarette (n = 49)	Total (n = 234)	F/χ^2^	p
	Mean (SD) or n (%)	Mean (SD) or n (%)	Mean (SD) or n (%)	Mean (SD) or n (%)		
**Behavioral disposition**						
Impulsivity (BIS)^a^	68.89 (9.22)	74.00 (9.14)	64.67 (8.55)	68.57 (9.39)	9.27*	.000
Aggression (BPAQ)	69.93 (17.97)	64.38 (14.76)	71.31 (17.49)	69.60 (17.58)	1.41	.247
Sensitivity to reward	12.18 (5.95)	10.54 (6.22)	12.31 (4.78)	12.03 (5.76)	0.99	.848
Sensitivity to punishment	10.61 (5.54)	10.35 (5.18)	10.12 (4.89)	10.48 (5.36)	0.17	.375
**Neuropsychological assessment**						
N-back memory						
Accuracy^b^					5.78	.004*
0-back	0.938 (0.142)	0.962 (0.062)	0.954 (0.075)	0.944 (0.124)		
1-back	0.768 (0.193)	0.739 (0.141)	0.869 (0.186)	0.786 (0.191)		
2-back	0.626 (0.229)	0.588 (0.253)	0.745 (0.174)	0.647 (0.227)		
Reaction time					.030	.971
0-back	448.188 (65.204)	450.411 (65.205)	459.412 (71.812)	450.785 ( 80.198)		
1-back	492.535 (102.897)	596.404 (104.423)	480.332 (98.658)	491.521 (102.013)		
2-back	582.334 (145.799)	562.321 (139.662)	572.398 (122.804)	578.030 (140.219)		

Bonferroni correction was used to adjust multiple comparisons. Tukey’s method was performed to test the post-hoc difference.

*p < .05/10 = .005.

^a^Polydrugs > Ketamine > Cigarette.

^b^Cigarette > Polydrugs, Ketamine.

**Table 4:** Psychiatric symptoms of study participants.

Variables	Ketamine (n = 159)	Poly drugs (n = 26)	Cigarette (n = 49)	Total (n = 234)	F/χ^2^	*p*
	Mean (SD) or n (%)	Mean (SD) or n (%)	Mean (SD) or n (%)	Mean (SD) or n (%)		
**Psychiatric symptoms, SCL-90R**						
Global severity index	1.37 (0.43)	1.41 (0.48)	1.57 (0.58)	1.42 (0.48)	3.24	.041
Somatization	14.53 (4.86)	15.35 (5.83)	16.18 (5.75)	14.97 (5.19)	1.99	.139
Obsessive–compulsive	15.32 (5.10)	15.76 (6.29)	18.14 (7.23)	15.96 (5.83)	4.55	.012
Interpersonal sensitivity	12.16 (4.28)	12.52 (4.80)	14.61 (7.18)	12.72 (5.15)	4.38	.014
Depression	18.29 (7.14)	18.62 (7.35)	21.10 (9.78)	18.92 (7.84)	2.45	.088
Anxiety	13.39 (4.94)	13.88 (5.29)	14.94 (5.49)	13.77 (5.11)	1.74	.179
Hostility	8.38 (3.41)	7.96 (2.39)	9.43 (4.28)	8.55 (3.58)	2.08	.127
Phobic anxiety	8.38 (2.83)	9.35 (3.72)	8.82 (3.01)	8.60 (2.98)	1.53	.218
Paranoid ideation	8.32 (3.27)	7.96 (2.72)	9.08 (3.41)	8.44 (3.25)	1.35	.260
Psychoticism	13.25 (4.33)	13.19 (3.92)	15.80 (6.35)	13.78 (4.88)	5.53	.005

Bonferroni correction was used to adjust multiple comparisons.

*p < .05/10 = .005.

## Discussion and conclusion

To our knowledge, this study is one of the few published studies to comprehensively examine and compare the clinical and behavioral characteristics of individuals who use ketamine, use polydrugs, and smoke cigarettes. The main findings are that drug-related problems, impulsivity, and memory impairment were more severe in the Ketamine and Polydrug groups than in the Cigarette group. Almost all the ketamine- using patients smoked it rather than snorted it. More than 80% of them had a full-time job. Additionally, a few patients had been infected with HIV and diagnosed with LUTS. A study of 106 ketamine users in Taiwan found that 84% developed LUTS after using ketamine for an average of 24.67 months, based on self-reports^39^. We speculate that the low incidence of LUTS among ketamine users in our study was because few of them snorted it, whereas more than 60% of ketamine users in Li’s sample snorted it. Snorting ketamine has been reported to cause LUTS more frequently than smoking it, due to a higher amount of ketamine entering the circulatory system^39^. Another possibility is that polydrug use may exacerbate LUTS. In our study, 14% were polydrug users compared to more than 80% used ketamine combined with other drugs to participants in Li’s study^39^. Future study is needed to clarify if polydrug use may exacerbate LUTS.

### Drug-related problems

Our results show that the Polydrug group had the most serious drug problems, followed by the Ketamine group and the Cigarette group, suggesting a greater severity of drug-related problems among polydrug users in the population. Development of sensitization and tolerance have been observed in studies of repeated or high doses of ketamine, suggesting that ketamine is an addictive substance. For instance, in one study naïve healthy subjects reported greater liking of and wanting more ketamine following acute ketamine exposure; also, when ketamine was combined with other illicit substances, the severity of drug addiction was found to increase^40^. This issue is especially relevant to young adults, such as the patients in our study, because their brains are not fully mature. Unfortunately, there is no research on the interaction of ketamine with other substances on the developing brain.

### Memory task

Consistent with prior studies^19,41^, in our study ketamine use was associated with impairment of performance on a task tapping working memory. A single dose of ketamine has been previously found to induce a deficit in working memory, and frequent ketamine users exhibited profound impairment in both long-term and short-term memory^1^. We found that, compared to cigarette smokers, drug users performed poorly on the NBMT, indicating a decrement in working memory. However, some researchers have argued that poor performance on such tasks reflects impaired vigilance or poor attentional functioning following ketamine use^42^. In our study, differences in reaction times on the NBMT across the three groups were not significant, but response accuracy was significantly higher in smokers than in ketamine and polydrug users. The frequent errors suggest that working memory impairment rather than general psychomotor slowing was the cause of the poor performance. Given that ketamine is a NMDA receptor antagonist and that these receptors are essential to memory, it is reasonable to hypothesize that ketamine alters brain function. It is also interesting to note that the Polydrug group, which consumed multiple drugs over a 30-day period, had the worst performance on the NBMT, implying greater impairment of memory in heavy users of polydrugs. Consistent with our findings, those of Morgan et al. suggest that the deficit in memory is dose-dependent and that frequent ketamine users should exhibit greater memory deficit than infrequent users^43^. Although our findings support the hypothesis that cognitive damage is associated with ketamine use, more research is needed to discover whether the decrement in working memory is reversible following abstinence.

### Ketamine use, HIV, and LUTS

Of the multiple adverse effects stemming from chronic ketamine use that we found in our study, of greatest concern to us were the noticeable rates of LUTS and HIV infection. In our sample, 5.7% of ketamine users and 3.8% of polydrug users were diagnosed as HIV positive, whereas there were no such diagnoses among the cigarette smokers. In China, the prevalence of HIV infection among drug users is well above 0.1% of the general population^44^. Correlational studies show that drug use, particularly use of methamphetamine, is strongly related to a higher risk of contracting a sexually transmitted disease^45^. Likewise, Lau et al. noted that users of substance engaged in sexual activities subsequent to drug intake, which put them at greater risk of contracting HIV^46^. The authors claimed that individuals who engage in high-risk drug use behavior are more likely to engage in other forms of risky behavior, such as unprotected sex, while under the influence of the drug. Drugs such as ketamine produce cognitive side effects such as impairment of judgment, further facilitating risk-taking behavior and exposure to individuals with HIV^46^. In addition, high-risk activity such as unprotected sex work is a common way to pay for illicit drugs^47^.

Another major physical harm associated with ketamine use is the development of lower urinary tract symptoms (LUTS), which can be long-lasting^1^. In our study, 6.9% of ketamine users and 11.5% of polydrug users reported altered micturition patterns. A study conducted in the UK found that 30% of ketamine users reported urinary tract symptoms and nearly half of frequent users needed medical treatment for urinary cystitis^48^. In a study conducted in Hong Kong, only a third of the cases resolved following the cessation of ketamine use, while the remaining patients experienced either no change or even a worsening of their symptoms, suggesting that the physical damage may be irreversible^49^. Cheung and colleagues concluded from their research that early cessation of ketamine is essential to improve urinary symptoms and quality of life^50^. The association between dosage, duration, and severity of symptoms is an under-explored topic in the ketamine literature and should be investigated in future studies.

### Impulsivity and ketamine use

As predicted, polydrug users were the most impulsive group in our study, and exclusive ketamine users were more impulsive than cigarette smokers. In the literature, substance use has consistently been associated with elevated impulsivity despite the large variability in the sample characteristics and the diversity of the measures of impulsivity. This association is expected, since impulsive individuals are more receptive to the reinforcing effect of substance abuse and have little regard for future negative consequences^51^. Dawe and colleagues hypothesized that the relationship between personality trait and drug use is bi-directional, in that impulsivity increases vulnerability to drug use, and chronic drug use weakens decision making so that quitting becomes more difficult^51^. The relationship between drug use and certain personality traits is well documented in the literature, yet little attention has been paid to ketamine in this regard. In our study, ketamine users showed greater impulsivity than cigarette smokers, reflecting an increased risk of continued drug use leading to addiction. For example, the highest scorer on the BIS-11 in our study reported engagement in multiple kinds of highly dangerous behavior, including polydrug use. Based on the theory of Dawe et al.^51^, it is plausible that impulsive traits predispose people to early drug experimentation, and the neural adaptation of the reward centers in the brain to chronic drug exposure reinforces further polydrug use and engagement in other kinds of high-risk behavior.

### Study limitations

The present study had several shortcomings. First, the representativeness of the sample was limited since participants in the Cigarette group were recruited by convenience sampling, and the size of the Polydrug group was modest. A larger and randomly selected sample would have been more representative. On the other hand, the three groups were similar with respect to demographic variables; thus, age, gender, and education, at least, were well matched. Second, the drug- and sex-related scores were based on participants’ retrospective estimates and we could not investigate the objectivity of these self-reports. Lastly, although we demonstrated several statistically significant associations between variables, the relationships were subject to a number of confounds, and we cannot address the direction of causation. For instance, we cannot determine whether impulsivity is a predisposing factor for chronic drug use or a consequence of it.

## Conclusion

In this study we uncovered a long list of clinical characteristics of chronic ketamine users, and we confirmed cognitive and physical damage associated with ketamine misuse. Importantly, we found that ketamine users were characterized by a noticeable rate of diagnosed HIV infections and of lower urinary tract symptoms. Polydrug use was associated with severe drug-related problems and high impulsivity. Cigarette smokers performed significantly better than drug users on the N-back Memory Task, which taps short-term and working memory.

We conclude from these findings that educational programs should include information on the cognitive and physical harm that can arise from Ketamine use, so that users can become aware of these detrimental effects. Additionally, our findings highlight specific groups of individuals that should be targeted for preventive interventions.
